# Local bi-fidelity field approximation with Knowledge Based Neural Networks for Computational Fluid Dynamics

**DOI:** 10.1038/s41598-021-93280-y

**Published:** 2021-07-14

**Authors:** Nick Pepper, Audrey Gaymann, Sanjiv Sharma, Francesco Montomoli

**Affiliations:** 1 UQLab, Department of Aeronautics, Imperial College London, SW7 2AZ London, UK; 2 Airbus UK, Filton, Bristol, BS34 7PA UK

**Keywords:** Aerospace engineering, Computational science

## Abstract

This work presents a machine learning based method for bi-fidelity modelling. The method, a Knowledge Based Neural Network (KBaNN), performs a local, additive correction to the outputs of a coarse computational model and can be used to emulate either experimental data or the output of a more accurate, but expensive, computational model. An advantage of the method is that it can scale easily with the number of input and output features. This allows bi-fidelity modelling approaches to be applied to a wide variety of problems, for instance in the bi-fidelity modelling of fields. We demonstrate this aspect in this work through an application to Computational Fluid Dynamics, in which local corrections to a velocity field are performed by the KBaNN to account for mesh effects. KBaNNs were trained to make corrections to the free-stream velocity field and the boundary layer. They were trained on a limited data-set consisting of simple two-dimensional flows. The KBaNNs were then tested on a flow over a more complex geometry, a NACA 2412 airfoil. It was demonstrated that the KBaNNs were still able to provide a local correction to the velocity field which improved its accuracy. The ability of the KBaNNs to generalise to flows around new geometries that share similar physics is encouraging. Through knowledge based neural networks it may be possible to develop a system for bi-fidelity, computer based design which uses data from past simulations to inform its predictions.

## Introduction

Computational simulations are playing an increasingly important role in engineering design, reducing the quantity of physical testing required by using simulations to model the performance of new designs. In order to produce designs that are reliable, the parametric uncertainties associated with the design variables must be taken into account. Uncertainty Quantification (UQ) typically involves propagating the parametric uncertainties associated with the inputs of a computational model in order to determine the effects of these uncertainties on the designs performance (see, e.g.^1^). On the other hand, reliability based design optimisation (RBDO) algorithms seek to find the design which minimises a cost function subject to probabilistic constraints^2^. Both UQ and RBDO algorithms rely on repeated simulations of a design for varying values of the design variables. A single simulation may be time consuming, for instance a single Computational Fluid Dynamics (CFD) simulation can take several hours to complete due to the mesh size and the complexity of the geometries studied. Consequently, repeated model evaluations can place an overwhelming demand on potentially limited computational resources.

An attractive strategy for mitigating the computational cost of these algorithms is to supplement the results of the most accurate models of a system, referred to as high-fidelity models, with models that are computationally less expensive. These low-fidelity models may have simplified physics, a coarser meshing or less detailed geometries and as a consequence are not as accurate. A popular area of research has been in developing multi-fidelity methods that can leverage relatively scarce high-fidelity data with low-fidelity data that allows trading accuracy with rapidity of results.

A particularly popular example of a multi-fidelity method is co-kriging^3,4^. An auto-regressive model is constructed to combine datasets of multiple fidelties, with the outputs of the model treated as a realisation of a Gaussian random variable. A Markov property is assumed: the coarse models cannot add additional information at locations where high-fidelity data is available. Co-kriging has been used widely among many disciplines, however, there are a number of drawbacks that make its application to industrial problems challenging. Training the kernel involves repeated inversions of the correlation matrix. This matrix scales with the size of the training dataset, which can become intractable for large datasets^5^. Secondly, the kernel is sensitive to the choice of kernel function, the choice of which is not trivial in high dimensional problems^6^. Conversely, neural methods have proved efficient for high dimensional meta-modelling^7,8^. However, while neural networks can act as powerful approximators, they are purely data-driven and function as black box models. Predictions made by neural networks are often criticised due to this lack of transparency^9,10^. For these reasons there is interest in developing deep neural networks which incorporate physical knowledge of a system^11^.

**Figure 1:**
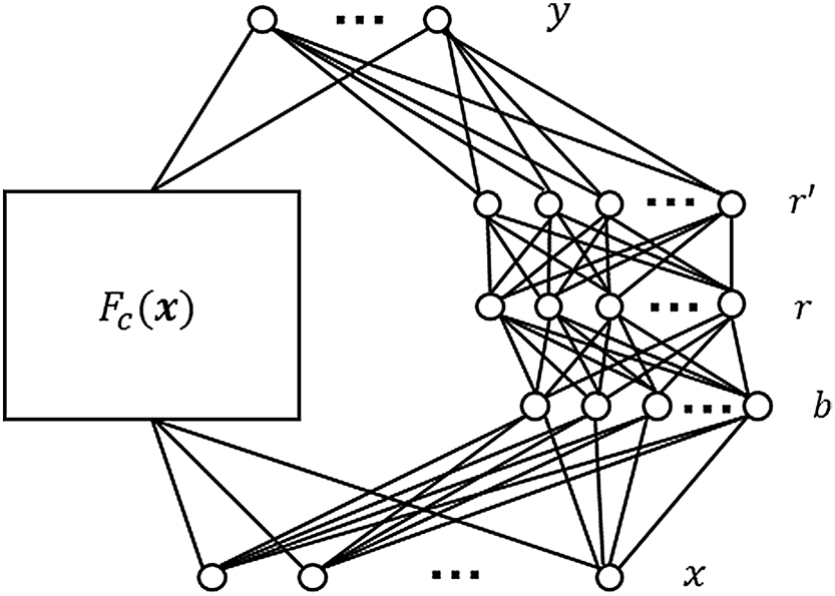
Architecture of a KBaNN for bi-fidelity modelling. A correction is made to the outputs ($\mathbf{y }$) of a coarse, low-fidelity model $F_c(\mathbf{x })$ by a network consisting of boundary neurons (*b*), region neurons (*r*), and normalised region neurons ($r'$) for an input $\mathbf{x }$ .

Knowledge based neural networks use prior knowledge of a system to inform their predictions. In the original formulation for a hybrid learning system proposed in Towell and Shavlik (1994) the prior knowledge is encoded in the network in the form of symbolic rules which determine the neural network structure and the initialisation of its weights. It was demonstrated that the performance of a classifier could be improved by incorporating these “domain theories”, especially if the data used to train the classifier was limited^12,13^. An alternative formulation for KBaNNs was later introduced in Wang and Zhang (1997) in which prior knowledge is embedded in a neural network in the form of a “knowledge layer” consisting of empirical functions^14^. Later works replaced the empirical functions with a low-fidelity model, producing a neural network for bi-fidelity modelling^15,16^. The general architecture for a KBaNN capable of bi-fidelity modelling is illustrated in Fig. 1, in the figure $F_c(\mathbf{x })$ refers to an evaluation of the coarse model, which is modified at the output layer by the neural network.

The high computational cost associated with individual simulations make CFD simulations a popular application in the multi-fidelity modelling literature. There are two approaches to defining a low-fidelity CFD simulation: the first approach is to alter the physics of the model so that it is cheaper to evaluate. For instance, Direct Numerical Simulation (DNS) is a high fidelity technique for modelling turbulent flows. The Navier-Stokes equations are solved at every length scale, allowing the turbulence to be completely resolved and hence providing complete knowledge of the flow. The drawback to the method is that it is very computationally expensive, with each simulation typically taking a number of days to run^17^. A number of works in the literature leverage a small set of DNS results with Reynolds-Averaged Navier-Stokes (RANS) simulations, which are less accurate due to simplifications in the turbulence closure but also cheaper to evaluate^18–20^. An alternative approach is to use the same model with meshes of varying coarseness in order to create low-fidelity surrogates. Examples of such an approach have been used in the literature for the design of a transonic compression rotor in Shapar et al (2011)^21^ and in Shah et al (2015)^22^ for airfoil design. This approach is similar to multi-scale modelling, where a relatively coarse model used to model the entire domain can be informed by more accurate models of a sub domain (see, e.g.^23,24^).

In this work a local Navier-Stokes approximator is used to correct for mesh effects in CFD simulations introduced by a coarse mesh. The approximator is a KBaNN that has been trained using a dataset of high-fidelity and low-fidelity CFD data. A crucial distinction between this work and the multi-fidelity modelling literature is that the multi-fidelity approximator is tested on data harvested from a flow around a geometry that is not the same as the geometry that was used to train it. In the test case presented here, the approximator is trained on a dataset comprising simulations of a two-dimensional channel flow and flow over a converging channel before being tested on a flow across a NACA 2412 airfoil. By learning the local corrections that must be made to the velocity field due to the coarseness of the mesh for a simple geometry, the system can generalise to flows around more complex geometries that share similar physics. As computational based design becomes an increasingly important part of designing new products, data-bases of simulation results will begin to accumulate. This paper presents a framework by which these databases may continue to add value, by training a multi-fidelity system that can be used to inform future designs.

## Methods

### Knowledge based neural networks (KBaNNs)

Knowledge Based Neural Networks are a bi-fidelity machine learning architecture that allow the outputs of a computationally inexpensive, but less accurate coarse model, $F_c(\mathbf{x })$, to be augmented by the predictions of a neural network. This correction takes the form of an additive correction applied to $F_c(\mathbf{x })$ in the output layer of the KBaNN. Having been trained using a dataset comprising outputs of a high-fidelity model or experiment, $F_e(\mathbf{x })$, the KBaNN corrects the outputs of the coarse model to emulate the output of $F_e(\mathbf{x })$. The generalised KBaNN architecture is illustrated in Fig. 1. The architecture is based on the KBaNN proposed in Wang et al (1997) but adapted for bi-fidelity modelling. The formalism has also been modified to produce an additive rather than multiplicative correction to $F_c(\mathbf{x })$. It also now incorporates $L^2$ regularisation. The motivation for both of these modifications is to improve the interpretability of the network; an additive correction to $F_c(\mathbf{x })$ makes the contribution of the neural network to the estimation easier to quantify. Similarly, we use regularisation to penalise network complexity that produces deviations from $F_c(\mathbf{x })$.

The neural architecture consists of five layers: input layer *x*, boundary layer *b*, region layer *r*, normalised region layer $r'$, and output layer $\mathbf{y }$. Neurons in the boundary layer are related to the KBaNN inputs through the vector of parameters $\mathbf{v }$ associated with each of the $n_b$ boundary neurons:

1 $$\begin{aligned} b_i=\mathbf{v }_i^T\mathbf{x },\quad i=1,2,...,n_b. \end{aligned}$$

This is a generalised formulation for the boundary neurons in which linear boundaries are assumed. It is noted in Wang et al (1997) that there is the potential to include problem specific boundary functions if these are known. Neurons in the region layer are evaluated in a similar fashion to the hidden layers of a Multi-Layer Perceptron (MLP), with weighted connections between the neurons in the region layer and boundary layer:

2 $$\begin{aligned} r_i=\prod _{j=1}^{n_b}\sigma (\alpha _{ij}b_j+\theta _{ij}), \quad i=1,2,...,n_r, \end{aligned}$$

where $\sigma (.)$ refers to a sigmoid activation function and $n_r$ the number of region neurons. The parameters $\alpha _{ij}$ and $\theta _{ij}$ refer to the weight and bias in the connection between the ith region neuron and jth boundary neuron. Rational function based neurons^25^ are used in the normalized region layer to normalize the region layer outputs:

3 $$\begin{aligned} r'_i=\frac{r_i}{\sum _{j=1}^{n_r}r_j}, \quad i=1,2,...,n_r. \end{aligned}$$

Finally, an additive correction with second order neurons^26^ is applied to the outputs of the coarse model in the output layer:

4 $$\begin{aligned} \mathbf{y }_j=\beta _jF_{cj}(\mathbf{x })+\sum _{i=1}^{n_r}\rho _{ij}r_i'+\beta _{j0}, \quad j=1,2,...,n_y, \end{aligned}$$

where $F_{cj}$ refers to the jth output of the coarse model. The parameter $\rho _{ij}$ weights the contribution of the ith region to the jth output neuron. The KBaNN is trained using a dataset that includes *P* evaluations of the fine model and the corresponding coarse model evaluations: $[\mathbf{x }_i,\; F_e(\mathbf{x }_i),\; F_c({\mathbf{x }_i})],\; i=1,...,P$. The error in the KBaNN prediction for the pth element of the training set is given by:

5 $$\begin{aligned} \epsilon =\frac{1}{2}\sum _{j=1}^{n_y}(\mathbf{y }_{pj}-F_{ej}(\mathbf{x }_p))^2+\lambda \Phi , \end{aligned}$$

i.e. the sum of the mean square error (MSE) and a $L^2$ regularisation term which is dependent on the complexity of the KBaNN:

6 $$\begin{aligned} \Phi =\sum _{i=1}^{n_b}\left\| \mathbf{v }_i\right\| _2^2+\sum _{i=1}^{n_r}\sum _{j=1}^{n_b}\alpha _{ij}^2+\sum _{i=1}^{n_r}\sum _{j=1}^{n_b}\theta _{ij}^2+\sum _{i=1}^{n_r}\sum _{j=1}^{n_y}\rho _{ij}^2+\sum _{j=1}^{n_y}\beta _{j0}^2+\sum _{j=1}^{n_y}(1-|\beta _j|)^2. \end{aligned}$$

The parameter $\lambda$, the regularisation constant, weights the contribution of the model complexity term to the prediction error. Penalising model complexity in this way is intended to produce a more parsimonious model. Whilst regularisation techniques in MLP’s typically act to force the network’s prediction to zero, in the case of the KBaNN the regularisation is instead used to punish predictions that deviate from the outputs of the coarse model. The KBaNN is trained through back-propagation using gradient descent optimisation. In this paper the adagrad algorithm for gradient based optimisation is used. Employing an adaptive learning rate, the magnitude of the updates made to the KBaNN parameters at each iteration is tailored to the frequency with which the features in the training set occur and is well suited for sparse datasets^27^. Incorporating information from the derivatives in the formulation for the learning rate ensures that the learning rate is monotonically decreasing and does not need to be manually tuned. More details on the error back-propagation, including the derivatives for the prediction error and Adagrad, may be found in Appendix A. An open source code for implementing the KBaNN is available in MATLAB^28^ and Python^29^.

### KBaNN architecture for a local Navier-Stokes approximation

In this work a KBaNN is used to perform a local correction to the results of a low-fidelity CFD simulation of a two-dimensional laminar flow. The KBaNN is trained using data taken from CFD simulations of a flow at two levels of meshing: a fine mesh which has fully converged to a solution (i.e. the mesh quality no longer affects the solution) and a coarser, more inaccurate mesh which is less computationally expensive to run. Having learnt the discrepancies between the high and low-fidelity mesh the KBaNN can then be employed to make corrections to CFD simulations of flows with similar physics.

**Figure 2:**
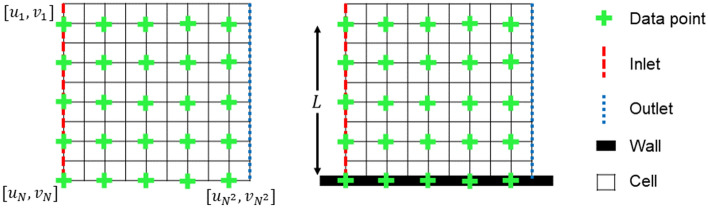
Schematic of the $N\times N$ sample grids used for the local Navier-Stokes approximation. There are two configurations for the grids: in the freestream or near a wall with a boundary layer present. Velocity data from the inlet of the grids were used as an input to the KBaNN, which estimated the velocities for the entire sample grid .

Local velocity data is extracted from each of the simulations by superimposing $N\times N$ sample grids on the flow and evaluating the velocity point at each data point. This is illustrated in Fig. 2, where a grid of data points is superimposed over a square mesh. For a two-dimensional flow with no separation there are two configurations that these sample grids may take depending on whether a wall is present. A KBaNN is then trained for each of these to situations i.e. a KBaNN trained for sample grids in the free-stream and a separate KBaNN trained for sample grids in the boundary layer, with a row of points inside a wall. The inputs of the KBaNN are the horizontal and vertical velocities along the ‘inlet’ side of the sample grid and the outputs the horizontal and vertical velocities of the entire sample grid. The output dimensions therefore scale quadratically with *N*, which demonstrates why it is advantageous to use a neural method for the bi-fidelity modelling as the number of dimensions can be significant even for a relatively small sample grid. Note that the spatial location of the grid is not included in the training data. This allows the KBaNN to be used on sample grids in the same flow in different locations or on sample grids in flows with similar physics but different geometries.

In order to prevent the grid size, *L*, from impacting the results the velocity data is scaled by a factor $\kappa =\frac{L}{L^*}$, where $L^*$ is the characteristic length scale of the flow. The KBaNN is used to predict the normalised velocities $u^*=\kappa u$ and $v^*=\kappa v$.

**Figure 3:**
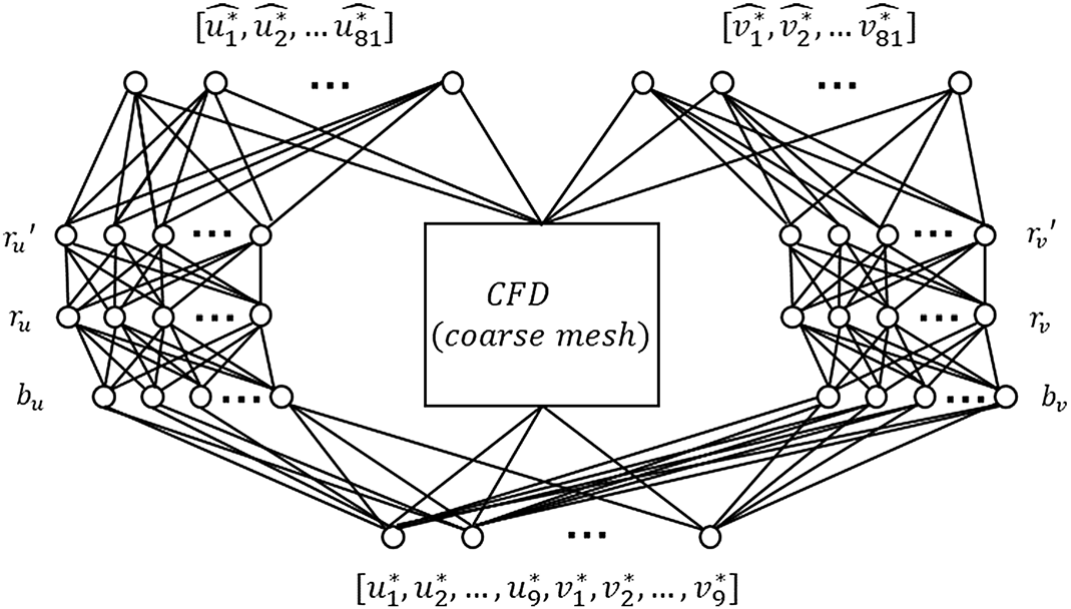
A modified KBaNN architecture was used for the local Navier-Stokes approximator. It was found that training a separate set of boundary and region neurons for each velocity component was more efficient (fewer neurons were required overall) than the general KBaNN architecture illustrated in Fig. 1 .

The architecture of the KBaNN used for local Navier-Stokes approximation is illustrated in Fig. 3 for a *N* = 9 sample grid. Note that this is a slight modification of the general KBaNN architecture illustrated in Fig. 1. The network is effectively split in half, with one portion of the network learning the corrections to the horizontal velocity and the other portion the vertical velocity correction. Splitting the network in this way was found to be more efficient as fewer neurons in total were required to fit the training data. This is a common technique that has been used in modular neural networks (see, e.g.^30^).

## Test case and results

The potential of the KBaNN architecture described above to act as a local Navier-Stokes approximator was demonstrated through two test cases. As described above, the system consisted of two KBaNNs: one of which was trained on data taken from the freestream and the other trained on grids located in the boundary layer. The KBaNNs were trained using data taken from seven simulations of a simple two-dimensional channel flow. In six of the simulations the channel walls were parallel, while in the seventh simulation one of the walls was inclined, creating a converging channel. The system was first validated using a channel flow simulation at a different Reynolds number before being applied to the more challenging case of a flow around a NACA 2412 airfoil. The intention was to train the system with a small dataset harvested from a relatively simple geometry and then to use it to make local Navier-Stokes estimations of a flow around a more complex geometry that shared similar physics.

For the test case of a channel flow at a different Reynolds number it was found that the system greatly improved the accuracy of the coarse mesh simulation. In the case of the airfoil the system led to an overall improvement in accuracy, despite the more complex geometry of the flow around the airfoil compared to the training data.

### Training and channel validation

**Figure 4:**
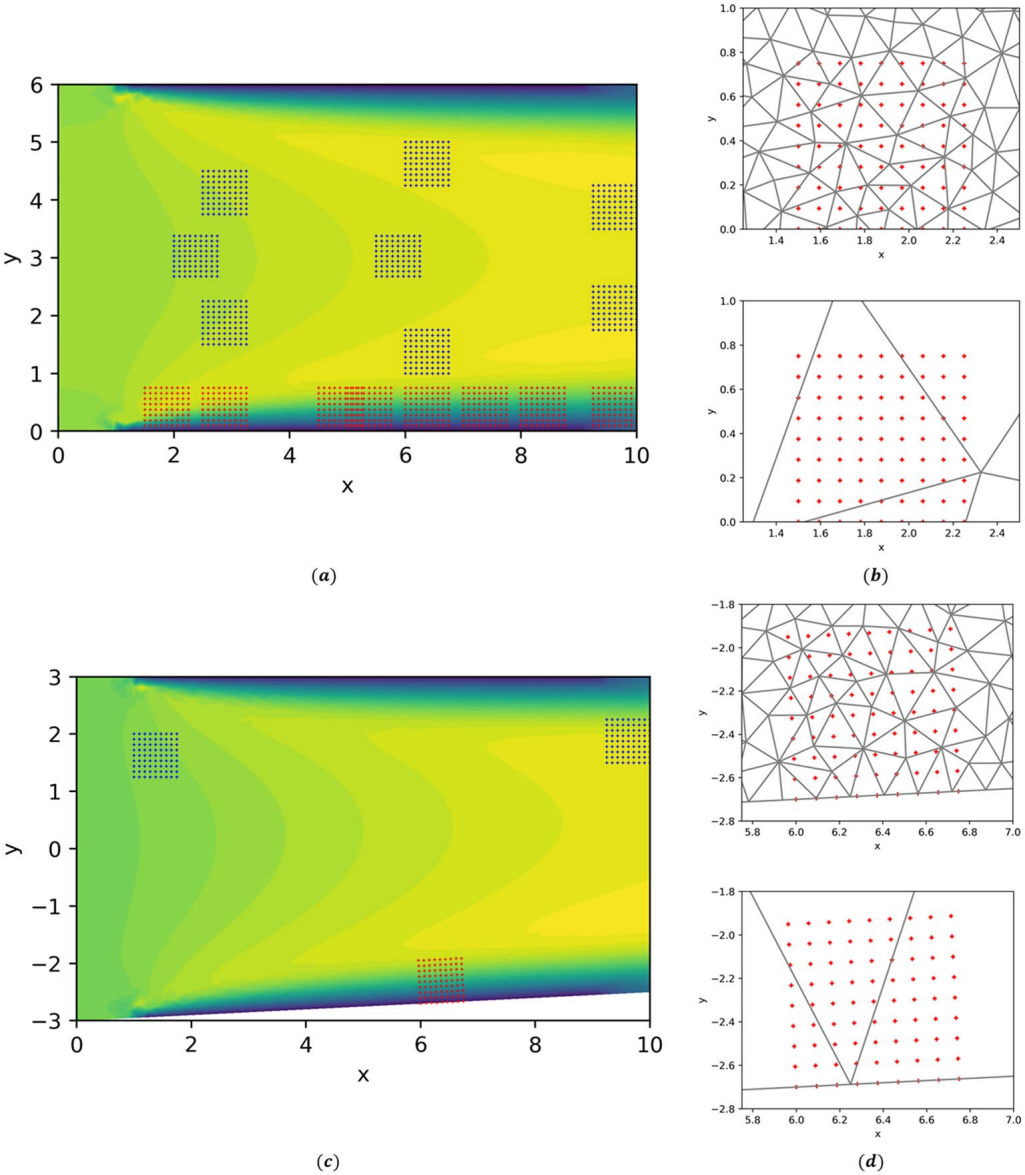
(**a**) A visualisation of the two-dimensional channel flow used to train the KBaNNs. The locations of the sample grids used to harvest velocity data are superimposed. Blue grids correspond to sample grids in the freestream, red to grids that bound the wall and capture the boundary layer. (**b**) Close ups of the mesh near the wall of the channel, with a sample grid superimposed. (**c**) A simulation of the flow over a slightly converging channel was included in the validation data set to prevent overfitting (**d**) the spatial resolution of the two meshes was kept the same.

Bi-fidelity CFD simulations of a laminar flow through a two-dimensional channel were used to train two KBaNNs so that the system could learn the discrepancies between the fine and coarse meshes. Figure 4a shows the two-dimensional channel flow and the locations of the sample grids which were used to train the KBaNNs. One KBaNN was trained using the blue sample grids located in the free stream, while the other was trained on the data from the red sample grids lying in the boundary layer attached to the channel wall. In Fig. 4b a sample grid is superimposed on plots of the coarse and fine meshes. As can be seen the spatial resolution of the fine mesh is significantly shorter than the length of the sample grid. It was chosen to be short enough to no longer affected the results of the simulation. The spatial resolution of the coarse mesh is around 8 times larger than that of the fine mesh. No-slip boundary conditions are applied to the boundaries at $y=0$ and $y=6$, an outflow at the boundary at $x=10$, and finally a uniform inflow was applied between $x=0$ and $x=1$. Simulations of the channel were run at varying values of the inflow velocity, *U*, for each mesh in order to generate a set of training data.

The training dataset was generated from the velocity data of the square, 9$\times$9 sample grids illustrated in Fig. 4a. As has been discussed above, the velocities were scaled to account for effects introduced by changing the size of the grid. Data was collected from grids of size $L=0.1, 0.5,$ and 0.75 so that these effects could be learned by the system.

A common problem in training neural networks is over-fitting the network to the training data. A strategy to prevent over-fitting is to hold aside some training data and use this as a validation dataset. Data was held aside from the channel configuration illustrated in Fig. 4a and also from a single simulation of the two-dimensional flow through a converging channel. This flow is illustrated in Fig. 4c. The spatial resolutions of the fine and coarse mesh were the same as with the other simulations, as can be seen in Fig. 4d. The bottom wall was inclined to give a 5% slope and the sample grids placed in different locations to the configuration in Fig. 4a. By including data from a flow around a different geometry in the validation set the KBaNNs were encouraged not to over-learn the discrepancies between the fine and coarse meshes. The following simulations were used for the training and validation datasets:

- **Training data**: Channel flow simulations at $Re=[300, 480, 600, 720, 900]$
- **Validation data**: Channel flow simulation at $Re=660$ and converging channel flow at $Re=480$

with data extracted from the sample grids locations displayed in Figs. 4a and 4c at three length scales. The KBaNN architecture illustrated in Fig. 3 was used. The sub networks were relatively small, with between 8-10 neurons in each layer and an equal number of neurons in the boundary and region layers. $9\times 9$ sample grids required each sub network to have 18 neurons in the input layer and 81 neurons in the output layer. Error back-propagation was used to train the KBaNNs, more details of which may be found in Appendix A. A gradient based optimisation algorithm was used to find values of the KBaNN parameter set $\Phi$ that minimised the mean squared error (MSE) of the validation dataset.

**Figure 5:**
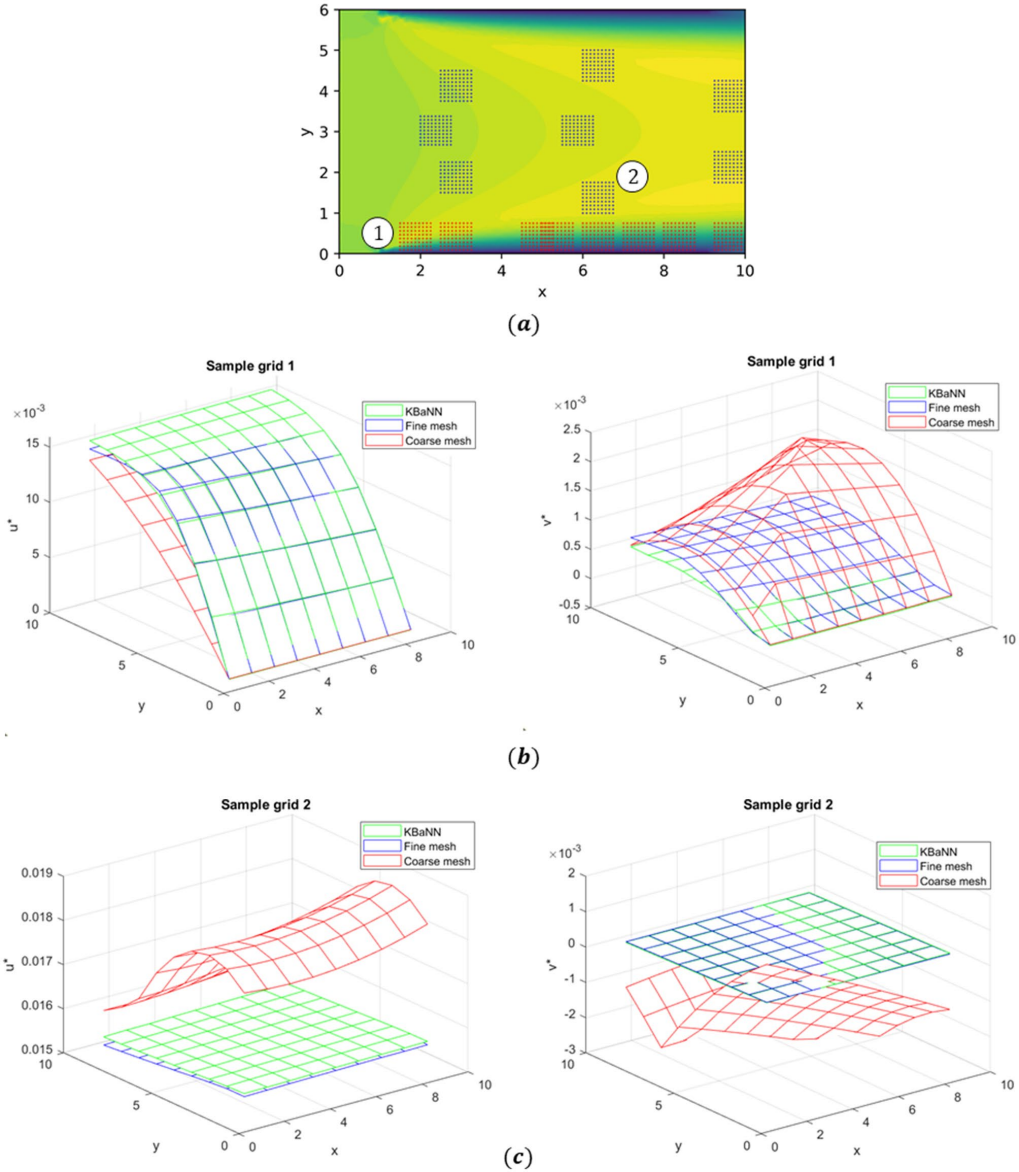
The performance of the KBaNNs was evaluated for a channel flow at $U=0.11$ms^-1^. (**a**) shows the locations of the two sample grids plotted. (**b**,**c**) the KBaNN predictions significantly improve the coarse mesh velocity field, while continuing to respect the no-slip boundary condition at the wall.

**Figure 6:**
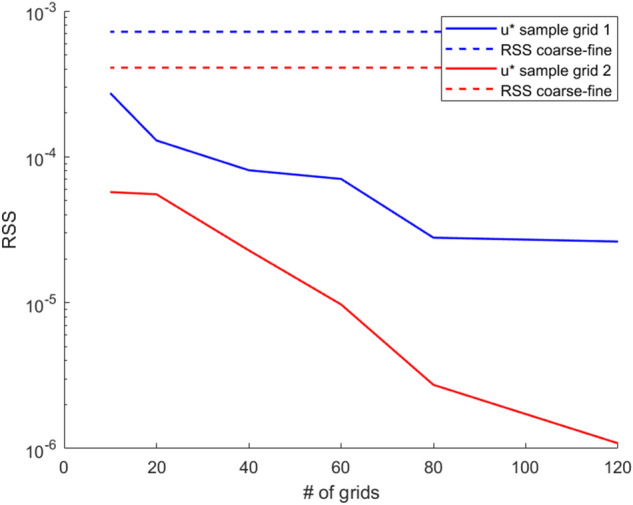
Plot of the performance of the KBaNNs as a function of the scarcity of the training data. Note that in all cases the RSS error of the KBaNNs was less than that of the coarse mesh.

Figure 5 displays the predictions of the KBaNNs for two sample grids in the channel simulation at $Re=660$ that was used in the validation dataset. These sample grids are labelled in Fig. 5a. In 5b–c the fine mesh solution is plotted against the estimations of the KBaNNs, with the coarse mesh solution included for comparison. As can be seen there is very good agreement between the fine mesh solution and the predictions of the KBaNNs. This agreement is reflected in Table 1, which tabulates the Residual Sum of Squares (RSS) differences between the coarse mesh and fine mesh solutions and between the KBaNN estimations and the fine mesh solution. Figure 6 illustrates the performance of the system as a function of the number of grids included in the training data. The RSS error between the fine and coarse mesh solutions are included as a comparison, represented by the horizontal lines in the figure. Increasing the scarcity of the training data reduced the performance of the system, however, it was found that even the KBaNNs trained with a minimal amount of data were more accurate than the coarse mesh solution in isolation.

This test demonstrates that the system can give accurate predictions for a flow that shares the same geometry as the flow used to train it. As can be seen in Fig. 5 the additive corrections made by the relatively small KBaNN effectively act as a high-order transformation of the coarse mesh solution.

**Table 1:** The residual sum of squares (RSS) between the KBaNN predictions and the fine mesh for each of the sample grids pictured in Fig. 5.

Sample grid	Velocity component	RSS between coarse and fine mesh	RSS between KBaNN and fine mesh
1	u*	7.2058e-4	8.3983e-6
	v*	6.6582e-5	1.0856e-6
2	u*	4.0879e-4	9.2442e-7
	v*	2.5176e-4	1.2501e-8

The corrections made by the KBaNNs greatly improve the accuracy of the solution.

### Airfoil test case

Having demonstrated that the KBaNNs were capable of giving highly accurate estimations of the local velocity field for data harvested from a geometry identical to that used to train it, the system was tested on a test dataset harvested from a more complex, unfamiliar geometry. A laminar flow around a NACA 2412 airfoil at $Re=480$ was chosen as the test geometry. Estimations are made challenging in the boundary layer by the curvature of the airfoil and in the freestream by the displacement caused by the airfoil.

**Figure 7:**
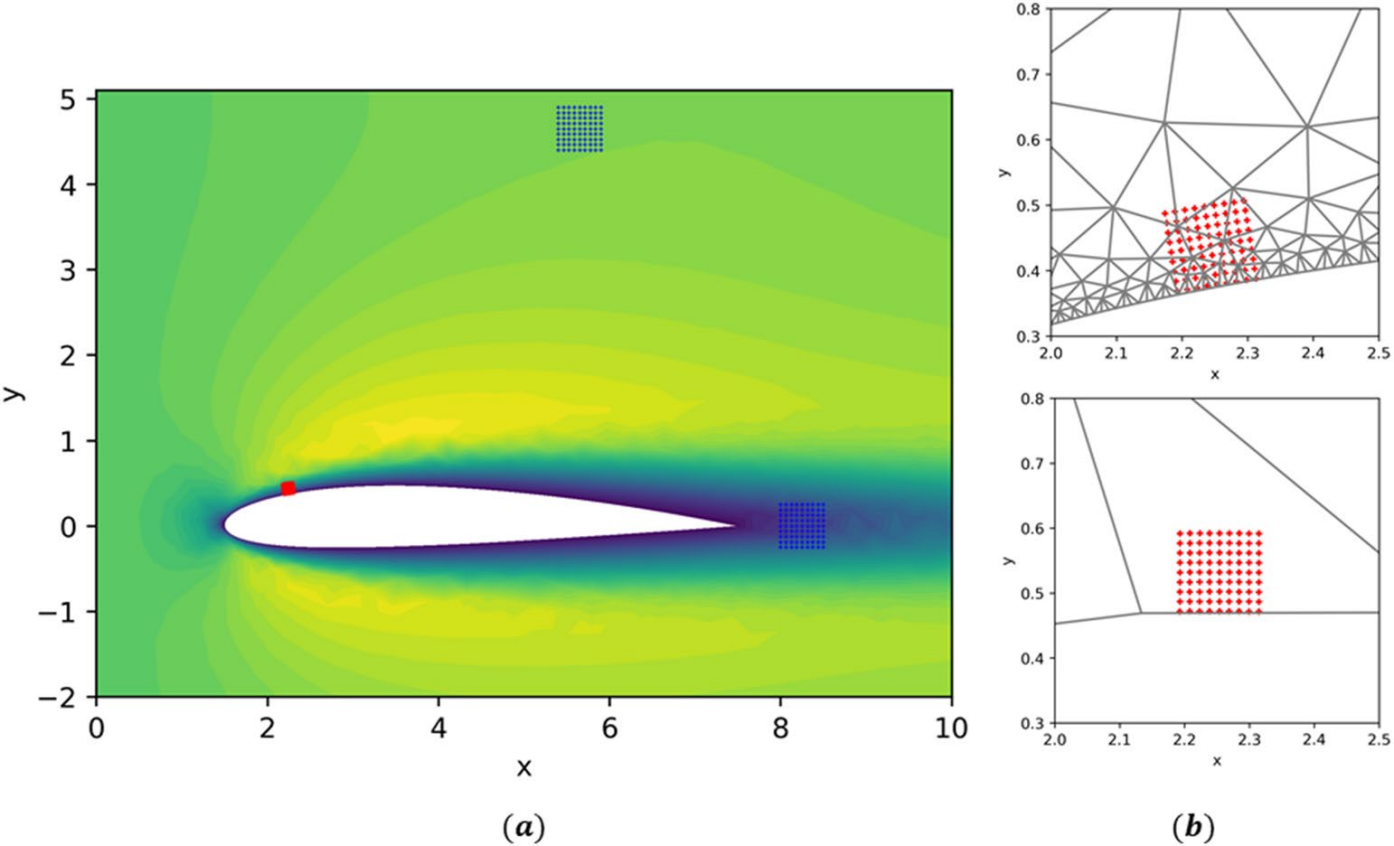
(**a**) Simulation results for the flow over a NACA 2412 airfoil at $Re=480$. Two sample grids were placed in the freestream and one on the top surface of the airfoil. (**b**) A closer view of the sample grid on the top surface of the airfoil.

The flow is visualised in Fig. 7a, with the locations of the sample grids superimposed. A sample grid was placed in the boundary layer on the top surface of the airfoil. Figure 7b displays this grid, superimposed on the two meshes. One challenge for the system is that the grid sits on a section that is locally flat in the coarse mesh, while the box in the coarse mesh curves to match the contours of the airfoil. The predictive power of the system is dependent on the data used to train it. Consequently, the sample grid in the boundary layer is situated upstream of the separation point, as the system has no experience of a separated boundary layer. Similarly, the sample grid placed in the freestream is located above the airfoil wake. The intention of the test case is to demonstrate that a system trained using data taken from simple flows can be used to make estimations in more complex flows, provided that the flow physics are sufficiently similar. Making predictions in separated boundary layers and wakes would require a more expansive training dataset, including flows with these features. To illustrate this point, we include a third sample grid in the wake of the airfoil.

**Figure 8:**
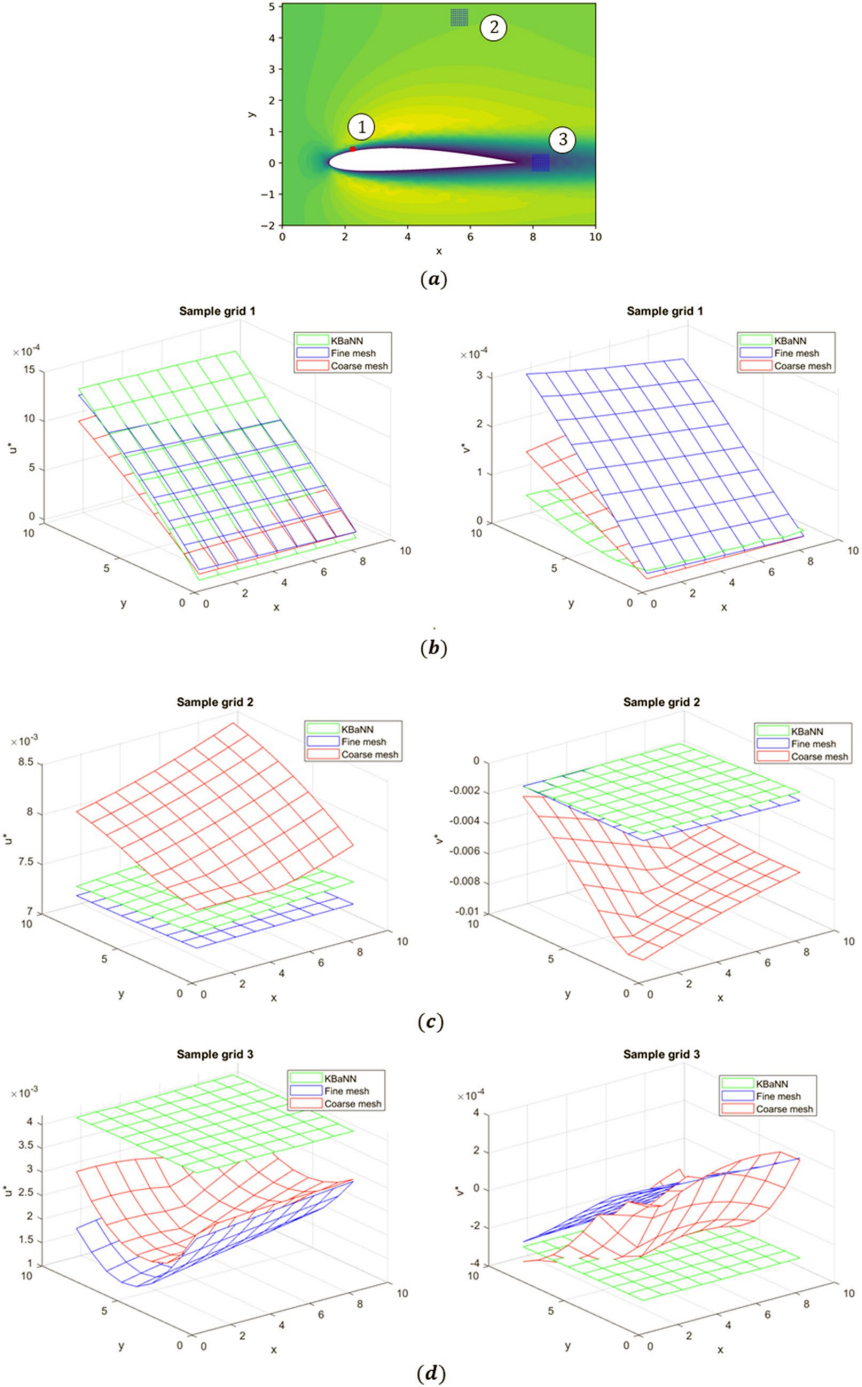
(**a**) the locations of the sample grids used to test the KBaNN. (**b**–**d**) A comparison of the predicted velocities in the sample grids from the KBaNN versus the fine mesh results. For comparison the velocities of the coarse mesh (which the KBaNN estimation is in part based on) are included.

Figure 8 displays the predictions of the KBaNNs for the sample grids around the NACA 2412 airfoil. The results of the fine mesh and coarse mesh CFD simulations are plotted for comparison. The RSS between the KBaNN predictions and the fine mesh results are tabulated in Table 2, along with the RSS between the results of the coarse and fine meshes for these grids. As with the channel test case, the freestream KBaNN is able to significantly improve the accuracy of the coarse mesh for the first two sample grids. The boundary layer KBaNN also leads to an overall increase in accuracy, although the system has difficulty estimating the vertical velocity of this grid due to the curvature. Nevertheless, this test shows that it is possible to use the KBaNNs on flows around different geometries to those in the training data provided the physics of the flow is sufficiently similar. The KBaNN cannot be applied indiscriminately however, as illustrated by sample grid 3, which is placed in the wake of the airfoil. The KBaNN approximation is inaccurate for this grid, as it is in a flow regime that was not included in the training data.

**Table 2:** The RSS between the KBaNN predictions and the fine mesh for each of the sample grids pictured in Fig. 8a.

Sample grid	Velocity component	RSS between coarse and fine mesh	RSS between KBaNN and fine mesh
1	u*	1.618e-6	3.1175e-7
	v*	4.1606e-7	1.713e-6
2	u*	5.2534e-5	2.0391e-6
	v*	1.9255e-4	9.094e-6
3	u*	4.1163e-5	4.3414e-4
	v*	2.6833e-6	1.0288e-5

For comparison the RSS between the coarse and fine mesh is included. As can be seen, the KBaNN improves significantly on the fine mesh prediction, provided the sample grid is within the training data.

## Conclusion

A neural based approach for bi-fidelity modeling, the KBaNN, has been introduced in this paper. An advantage of the method is that it can scale easily with the number of input and output features. This allows bi-fidelity modelling approaches to be applied to a wide variety of problems, for instance in the bi-fidelity modelling of fields. In this paper, KBaNNs were applied to a problem in CFD, in which mesh effects introduced variations in the estimation of a velocity field. KBaNNs were trained using data harvested from a limited number of runs of a simple flow, before being applied to a flow over a more complex geometry, but with similar physics. It was demonstrated that the KBaNNs could still produce a more accurate estimate of the flow in the freestream and near the top surface of the airfoil than could be provided by only using a coarse mesh, despite the KBaNNs not having ‘seen’ the airfoil geometry in training.

Allowing the KBaNNs to be informed by the low-fidelity model of the system helps to address the criticism of neural networks that they are black boxes which are completely data-driven. This criticism has implications for the trustworthiness and interpretability of neural network predictions. Interpretability motivates our use of the additive correction in the KBaNN architecture, rather than a multiplicative one. A further extension to the KBaNN architecture that would help address the issue of trustworthiness would be to incorporate a metric for the predictive uncertainty in the network, analogous to the kriging variance in Gaussian Processes.

The KBaNN architecture provides a framework for bi-fidelity modelling in which problems with multiple output features can be efficiently handled. For this reason the KBaNN is a natural choice for the bi-fidelity modelling of fields. The particular application to the velocity field predicted by CFD in this work offers a framework through which data-bases of simulation results for old designs can be used to inform the simulations of new designs. The KBaNNs were tested on a new geometry, with sample grids that were of a different size to those used to train them, which helps convince us that the KBaNNs make estimates that are to some extent informed by the physics of the model, rather than over-fitting patterns in the training data-set. In this study we developed a KBaNN architecture to correct mesh effects in a flow in the laminar regime, without separation. An interesting extension would be to diversify the training data available to the KBaNNs to allow it to make estimations for more complex flows, for instance flows in three dimensions or including wakes and separation. The system could then be expanded to include a number of KBaNNs, each trained on its own set of training data, with a classifier used to determine which KBaNN is suitable for a given regime.

## Supplementary information

Supplementary Information.
