# Supplementary material for: Local bi-fidelity field approximation with Knowledge Based Neural Networks for Computational Fluid Dynamics
Source: Sci Rep. 2021 Jul 14;11:14459. doi: 10.1038/s41598-021-93280-y (PMC8280202; doi:10.1038/s41598-021-93280-y)
Supplement: Supplementary file 1 — Supplementary Information. [file 41598_2021_93280_MOESM1_ESM.pdf]

Appendix A

As has been discussed above the KBaNN is trained through error back-propagation, with the network parameters determined through a gradient based optimisation algorithm. This required analytic expressions for the derivatives of the KBaNN prediction error, ε, with respect to the network parameters. Expressions for these derivatives in a KBaNN architecture were first derived in Wang et al (1997). However, the architecture presented here differs in that the correction applied is additive rather than multiplicative. A further difference is that the knowledge layer presented in the original paper have been replaced with a coarse model which is not necessarily analytic. As a consequence we do not adjust the parameters of the coarse model through back-propagation. In the KBaNN formulation presented here, there is only a single path for error back-propagation through the KBaNN which simplifies the formulae for the derivatives. Finally, the term added to the error term to penalise KBaNN predictions that deviate from the coarse model output mean that there is an additional term to the error derivatives. The error function derivative for the output layer may be derived from equation 5:

$$\frac{\partial \varepsilon}{\partial y_j} = y_j - F_e(\mathbf{x}), \quad j = 1,2,...,n_y. \tag{1}$$

Derivatives of the error function for the connections between the output neurons, the coarse model, and the normalised region neurons may then be derived from equation 4:

$$\frac{\partial \varepsilon}{\partial \beta_j} = (y_j - F_e(\mathbf{x}))F_{cj}(\mathbf{x}) + 2\lambda(\beta_j - 1), \quad j = 1,2,...,n_y, \tag{2}$$

$$\frac{\partial \varepsilon}{\partial \beta_{0_j}} = (y_j - F_e(\mathbf{x})) + 2\lambda\beta_{0_j}, \quad j = 1,2,...,n_y, \tag{3}$$

$$\frac{\partial \varepsilon}{\partial \rho_{ij}} = (y_j - F_e(\mathbf{x}))r'_i + 2\lambda\rho_{ij}, \quad i = 1,2,...,n_r, \quad j = 1,2,...,n_y. \tag{4}$$

The chain rule may then be employed to evaluate the gradients in the normalised region layer, the region layer, and the boundary layer respectively:

$$g_{r'_i} = \sum_{j=1}^{n_y} \frac{\partial \varepsilon}{\partial y_j}\frac{\partial y_j}{\partial r'_i} = \sum_{j=1}^{n_y}(y_j - F_e(\mathbf{x}))\rho_{ij}, \quad i = 1,2,...,n_r, \tag{5}$$

$$g_{r_i} = \sum_{j=1}^{n_r} \frac{\partial \varepsilon}{\partial r'_j}\frac{\partial r'_j}{\partial r_i} = g_{r'_i}\frac{1}{\sum_{k=1}^{n_r} r_k} - \frac{\sum_{j=1}^{n_r} g_{r'} r_j}{(\sum_{k=1}^{n_r} r_k)^2}, \quad i = 1,2,...,n_r, \tag{6}$$

$$g_{b_i} = \sum_{j=1}^{n_r} \frac{\partial \varepsilon}{\partial r_j}\frac{\partial r_j}{\partial b_i} = \sum_{j=1}^{n_r} g_{r_j} r_j (1 - \sigma(\alpha_{ji}b_i + \theta_{ji}))\alpha_{ji}, \quad i = 1,2,...,n_b, \quad j = 1,2,...,n_r. \tag{7}$$

Equation 2 may then be used, together with these derivatives to find the error function gradient for the region layer parameters:

$$\frac{\partial \varepsilon}{\partial \alpha_{ij}} = \frac{\partial \varepsilon}{\partial r_i}\frac{\partial r_i}{\partial \alpha_{ij}} = g_{r_i} r_i (1 - \sigma(\alpha_{ij}b_j + \theta_{ij}))b_j + 2\lambda\alpha_{ij}, \quad i = 1,2,...,n_r, \quad j = 1,2,...,n_b, \tag{8}$$

$$\frac{\partial \varepsilon}{\partial \theta_{ij}} = \frac{\partial \varepsilon}{\partial r_i}\frac{\partial r_i}{\partial \alpha_{ij}} = g_{r_i} r_i (1 - \sigma(\alpha_{ij}b_j + \theta_{ij})) + 2\lambda\theta_{ij}, \quad i = 1,2,...,n_r, \quad j = 1,2,...,n_b. \tag{9}$$

Finally, the derivatives for the parameters in the boundary layer may be calculated using equation 1:

$$\frac{\partial \varepsilon}{\partial \mathbf{v}_{j_i}} = g_{b_j}\mathbf{x}_i + 2\lambda\mathbf{v}_{j_i}, \quad i = 1,2,...,n_x, \quad j = 1,2,...,n_r. \tag{10}$$

Adagrad is a gradient based optimisation algorithm used to update the KBaNN parameters with a variable learning rate. The set of KBaNN parameters, Φ, are initialised with random values and the training set iterated through. At each iteration the KBaNN parameters are adjusted according to:

$$\Phi_{i,t+1} = \Phi_{i,t} - \frac{\eta}{\sqrt{G_{t_{ii}} + \tau}}\frac{\partial \varepsilon}{\partial \Phi_i}, \tag{11}$$

where G_t is a diagonal matrix, in which the element $G_{t_{ii}}$ contains the sums of the squares of the past error function gradients for the ith KBaNN parameter. The constant τ is a smoothing constant used to ensure numerical stability when the gradients are small. After each complete pass through the training data, the data is shuffled to prevent the KBaNN learning spurious patterns.
